# Islet **β**-Cell Mass Preservation and Regeneration in Diabetes Mellitus: Four Factors with Potential Therapeutic Interest

**DOI:** 10.1155/2012/230870

**Published:** 2012-08-05

**Authors:** Jose Manuel Mellado-Gil, Nadia Cobo-Vuilleumier, Benoit R. Gauthier

**Affiliations:** Pancreatic Islet Development and Regeneration Unit, Department of Stem Cells, CABIMER-Andalusian Center for Molecular Biology and Regenerative Medicine, Avenida Américo Vespucio, Parque Científico y Tecnológico Cartuja 93, 41092 Sevilla, Spain

## Abstract

Islet **β**-cell replacement and regeneration are two promising approaches for the treatment of Type 1 Diabetes Mellitus. Indeed, the success of islet transplantation in normalizing blood glucose in diabetic patients has provided the proof of principle that cell replacement can be employed as a safe and efficacious treatment. Nonetheless, shortage of organ donors has hampered expansion of this approach. Alternative sources of insulin-producing cells are mandatory to fill this gap. Although great advances have been achieved in generating surrogate **β**-cells from stem cells, current protocols have yet to produce functionally mature insulin-secreting cells. Recently, the concept of islet regeneration in which new **β**-cells are formed from either residual **β**-cell proliferation or transdifferentiation of other endocrine islet cells has gained much interest as an attractive therapeutic alternative to restore **β**-cell mass. Complementary approaches to cell replacement and regeneration could aim at enhancing **β**-cell survival and function. Herein, we discuss the value of Hepatocyte Growth Factor (HGF), Glucose-Dependent Insulinotropic Peptide (GIP), Paired box gene 4 (Pax4) and Liver Receptor Homolog-1 (LRH-1) as key players for **β**-cell replacement and regeneration therapies. These factors convey **β**-cell protection and enhanced function as well as facilitating proliferation and transdifferentiation of other pancreatic cell types to **β**-cells, under stressful conditions.

## 1. Diabetes Mellitus and ***β***-Cell Regeneration

The global incidence of Diabetes Mellitus (DM) has increased alarmingly in the past ten years, becoming one of the most common chronic diseases. It is estimated that this disorder will affect 552 million people by 2030 (http://www.idf.org/media-events/press-releases/2011/diabetes-atlas-5th-edition). Changing lifestyle leading to reduced physical activity and increased obesity has been pointed as the major culprit for this increase. DM is a group of metabolic diseases characterized by hyperglycemia due to defects in insulin secretion by pancreatic *β*-cells, insulin action on target tissues, or both [1]. Based on its etiology, DM has been classified into four main groups [1]; (1) Type 1 DM (T1DM) that results from lack of insulin production due to selective autoimmune destruction of pancreatic *β*-cells; (2) Type 2 DM (T2DM) caused by a combination of insulin resistance in the main target tissues (liver, muscle, and fat) and inadequate compensatory insulin secretion response by *β*-cells; (3) other specific type of diabetes which includes the genetic defects of the *β*-cell; (4) gestational diabetes mellitus (GDM), characterized by glucose intolerance with onset or first recognition during pregnancy.

Existing treatments for T1DM and long-term T2DM patients are primarily focused on insulin supplementation. However, despite the beneficial effects of insulin therapy on glucose homeostasis, poor patient implementation often leads to diabetic complications such as retinopathy and nephropathy as well as cardiovascular and cerebrovascular diseases [1]. The life-threatening side effects of poor insulin management expose the need for new therapeutic strategies to preserve or replenish the functional *β*-cell mass and consequently maintain glucose homeostasis. In this scope, phase II clinical trials performed in patients with recent-onset T1DM using anti-CD3 monoclonal antibodies that suppress the immune system showed significant preservation of *β*-cell function for at least 24 months and to decrease insulin requirements [2]. Despite these beneficial effects, this intervention alone does not restore normal glucose control highlighting the need for the development of alternative therapies aimed at preserving or replacing destroyed *β*-cells. In the past 10 years, islet transplantation has gained much attention as a cell replacement therapy for restoring the functional *β*-cell mass. Approximately 400 individuals have received allogeneic isolated islets since 1999 [3]. Unfortunately, the limited supply of islets from donors cannot meet the demand imposed by the ever-growing number of T1DM patients. Furthermore, a major hurdle has been the lack of durability of islet function resulting in only less than 50% of recipients with insulin independence two years after transplantation [4]. In addition to side effects produced by immunosuppression, several posttransplant events, such as instant blood mediated inflammatory reaction (IBMIR) and cytokine cascade, seriously affect the functionality of transplanted islets [5–8]. Nonetheless, the promising outcome of islet transplantation has prompted the search for alternative sources of *β*-cells. One such alternative has been differentiation of insulin-producing cells from embryonic and adult stem/progenitor cells. However, despite the high number of differentiation protocols described in the literature, none published to date have generated by exclusive *in vitro* differentiation sufficient numbers of insulin-producing cells meeting all essential criteria that define a functional *β*-cell [9].

A more recent and provocative alternative to cell replacement therapy for the treatment of DM has been the concept of *in vivo* regeneration to replenish the loss of *β*-cell mass. Basically, the ultimate goal of this strategy is to enhance the regenerative capacity of the pancreas. Studies in animal models and in humans have demonstrated that obesity-associated insulin resistance as well as increased insulin requirements during pregnancy is matched by a corresponding stimulation in insulin output through *β*-cell hyperplasia and hypertrophy [10]. Studies in the non-obese diabetic (NOD) mouse, an important animal model of experimental autoimmune diabetes, have confirmed the importance of *β*-cell replication and improved survival under pathophysiological conditions. It was shown that *β*-cell replication increases with the occurrence of islet inflammation during progression of DM. Nonetheless, by three months mice develop hyperglycemia as the autoimmune attack counteracts the attempt of *β*-cells regeneration by the organism [11]. Of note, in condition in which autoimmunity and islet inflammation are blunted using an anti-CD3 immune therapy, newly formed *β*-cells derived from preexisting ones are sufficient to normalized blood glucose levels [12]. These studies highlight a fundamental paradigm in the approach to treat DM. A combinatorial approach in which both regeneration and resistance to destruction are enhanced will need to be targeted in order to successfully regain a functional *β*-cell mass and maintain normoglycemia. A regenerative approach to DM is a matter of life and death. In this context, an array of growth factors and transcription factors have been shown to be involved in these regulatory pathways, providing an attractive perspective for the development of new therapeutic strategies to maintain adequate *β*-cell mass for stabilization or even improvement of glucose metabolism in DM patients. The question is which ones from the ever-growing list of factors will convey, alone or in combination, the best winning chance of survival and growth to *β*-cells? In this context we have selected 4 candidates, the hepatocyte growth factor (HGF), the growth inhibitory peptide or glucose-dependent insulinotropic polypeptide (GIP), the transcription factors Pax4, and the orphan nuclear liver receptor homolog-1 (LRH-1) as rising stars in the field of *β*-cell regeneration. These factors exert several beneficial actions on *β*-cells simultaneously such as conferring *β*-cell protection and enhancing proliferation. In addition, some of them have been shown to improve *β*-cell function under pathophysiological conditions. Thus, they are very attractive targets for that combinatorial approach to treat DM, in which *β*-cell mass is not only increased but also protected from death. These four factors could also be utilized in *β*-cell replacement therapies as a part of either pre- or postislet transplantation treatments.

This paper highlights recent advances pinpointing to the importance of these candidates in *β*-cell regeneration as well as to provide insight on how at the molecular level they merge towards similar targets involved in cell replication and survival. These may eventually be employed alone or in combination in an effort to increase islet mass, viability, and function in patients with DM.

## 2. The HGF/c-Met Signaling Pathway

 HGF is a heterodimeric protein that binds to the tyrosine kinase receptor c-met. Stimulation of c-met by HGF results in the activation of several downstream signal transduction pathways including PI3K/AKT, MAPK, and PKC [13]. Interestingly, HGF was also shown to induce Wnt-independent nuclear translocation of *β*-catenin subsequent to met-*β*-catenin dissociation in liver cells resulting in cell replication [14]. In this context, the HGF/c-met axis possesses potent mitogenic effect on various tissues such as liver, lung, kidney, spinal cord, heart, and skeletal muscle upon injury [15, 16]. HGF and c-met are also expressed in the pancreas, and levels are significantly increased in islets of gestating mice as well as in cytokine-treated islets. The latter suggests that the HGF/c-met signaling pathway may be crucial for islet adaptation in response to metabolic demands [17, 18]. Consistent with this premise, pancreas-specific ablation of c-met increased sensitivity of islets to cytokine-induced apoptosis *in vitro* through increased NF-*κ*B signaling and nitric oxide production. Treatment of c-met null animals with multiple low doses of STZ, a *β*-cell specific diabetogenic agent, resulted in increased islet chemokine secretion, higher degree of insulitis, and increased *β*-cell apoptosis as compared to that observed in wild type treated littermates. These inflammation features combined with *β*-cell death led to a reduced *β*-cell mass and higher incidence of hyperglycemia [18]. Similarly, pregnant mice lacking c-met in the pancreas displayed blunted *β*-cell replication with a concomitant increase in cell death leading to GDM. This defect was linked to reduced levels of the prolactin receptor as well as in levels of the transcription factor FoxM1, two important players in cell proliferation. A decreased nuclear translocation of STAT5 and upregulation of the replication inhibitor p27 were also detected in c-met-ablated islets [17]. These studies clearly identify the HGF/c-met signaling pathway as an important axis activated in conditions of metabolic stress in which islets require increased protection and proliferation. Thus, manipulation of this pathway may provide a new therapeutic venue for the treatment of DM. Consistent with this premise, adenoviral-mediated expression of HGF in nonhuman primate islets markedly improved function of transplanted islets. Indeed, only a marginal mass of islets expressing HGF was required to correct hyperglycemia in NOD-SCID mice as compared to the number of control islets needed to normalize blood glucose levels in the same animals. Furthermore, HGF-expressing grafted islets exhibited less apoptosis [19–21]. Although similar effects need to be established for human islets, these results are certainly encouraging and validate the usefulness of HGF for novel regenerative and replacement DM therapies.

In addition to its effect on proliferation and survival, HGF in combination with betacellulin was recently shown to induce *in vitro* transdifferentiation of pancreatic ductal endothelial cells (PDECs) into *β*-cells [22, 23]. Approximately, 30% of HGF-treated PDEC cells transdifferentiated into insulin-producing cells. This percentage was increased to 60% when HGF was used in combination with betacellulin. The phenotype of these newly differentiated *β*-cells was very similar to that of mature *β*-cells, expressing near physiological levels of insulin and GLUT2. These cells also exhibited insulin secretion in response to glucose. Interestingly, transdifferentiation of these PDECs was dependent on the temporal and sequential expression of key endocrine development transcription factors such as PDX-1, Ngn3, NeuroD, and Pax4 [23]. Using specific inhibitors for the different signaling pathways activated in PDECs upon HGF treatment, Li et al. suggested that HGF induces PDECs differentiation into insulin-producing cells through the PI3K/AKT cascade [22]. Whether similar transdifferentiation can occur *in vivo* remains to be established. The latter will also be valuable to determine the potential mitogenic side effect of HGF on PDECs. Indeed, Li et al. found that HGF increased proliferation of PDEC through activation of the MEK1/2-ERK1/2 pathway [22]. Thus, the balance between HGF-mediated proliferation and transdifferentiation into *β*-cells should be carefully investigated. Taken together, recent findings reinforce the value of HGF as a potential therapeutic agent that can increase islet number, viability, and functionality, three vital attributes for the treatment of DM through regenerative medicine. 

## 3. The Incretin GIP

GIP and glucagon-like peptide-1 (GLP-1) are two incretin hormones produced by the K and L-cells of the intestine, respectively. These incretins are secreted in response to food intake and potentiate glucose-induced insulin secretion from pancreatic *β*-cells in healthy individuals. Nonetheless, both peptides are rapidly degraded by dipeptidyl peptidase IV (DPP-IV) [24]. In view of their insulinotropic properties, major efforts have focused on developing either mimetics of incretins, which are resistant to DPP-IV or inhibitors of DPP-IV. Historically, GIP was rapidly discarded as a therapeutic target due to its impaired insulinotropic effect in T2DM patients. Nonetheless, recent evidence suggests that GIP may be a promising target for the preservation and regeneration of a functional *β*-cell mass in DM. Indeed, GIP was shown to be important for *β*-cell development as well as postnatal islet mass expansion and function. Transgenic mice expressing a dominant negative GIP receptor specifically in pancreatic *β*-cells displayed an early disturbance in pancreatic islet development with a severe reduction in the *β*-cell mass with a commensurate increase in *α*-, *δ*-, and PP-cells [25]. Adult transgenic animals had reduced number of islets and *β*-cells as compared to control littermates. Minimal postnatal islet expansion occurred due to a reduction in islet neogenesis. Intriguingly, islet proliferation and apoptosis were only slightly modified as compared to control mice [26]. Consistent with altered islet architecture, insulin secretion was impaired and animals developed early-onset DM [25].

In addition to its profound impact on development, GIP was also shown to increase mouse islet *β*-cell survival in response to high glucose and lipid levels (glucolipotoxicity) through activation of the PI3K/PKB pathway and downregulation of the proapoptotic factor Bax [27]. In addition, GIP stimulated expression levels of the antiapoptotic bcl-2 [28]. Studies using human islets revealed that GIP could partially block cytokine-mediated cell death clearly providing protective properties to this incretin. Interestingly, this antiapoptotic effect appeared to be conveyed by GIP-mediated increases in islet osteopontin [29]. Consistent with these *in vitro* data, administration of a DPP-IV-resistant analogue of GIP to rats blunted the adverse effects of streptozotocin on islet *β*-cell destruction and development of hyperglycemia. Furthermore, the GIP analogue also preserved *β*-cell mass in ZDF rats through decreased apoptosis [30]. *In vitro*, GIP was shown to stimulate proliferation of newborn rat islet *β*-cells, a mechanism potentially involving the activation of cyclin D1 [31]. Thus, GIP analogues resistant to DPP-4 cleavage may represent a promising new class of therapeutic compounds with properties to enhance as well as to preserve the critical *β*-cell mass required to maintain normoglycemia in DM patients.

## 4. The Islet-Enriched Transcription Factor Pax4

Pax genes encode a family of transcription factors that are key regulators of tissue development and cellular differentiation in embryos acting to promote cell proliferation, migration and survival. In the pancreas, Pax4 was shown to be essential for the generation of islet cell progenitors and subsequent *β*- and *δ*-cell maturation during embryogenesis [32, 33]. Although detectable, the expression of the transcription factor in adult islet *β*-cells was found to be low as compared to its embryonic expression [34, 35]. In contrast, aberrantly high expression levels of Pax4 were detected in human insulinomas as well as lymphomas [36, 37]. A distinctive attribute of Pax4 is that mutations and polymorphisms in this gene have been associated with both T1DM and T2DM in several ethnic populations [38]. Taken together, these unique characteristics strongly indicate a vital role of Pax4 not only during development but also in survival and/or maintenance of cell mass in mature islets. Consistent with the notion that Pax4 expression may be important for *β*-cell adaptation, *in vitro* studies performed in human islets demonstrated that glucose as well as growth factors such as betacellulin, activin A, and GLP-1 increased Pax4 mRNA levels [35]. Furthermore, Pax4 levels were also found elevated in islets derived from T2DM patients correlating with hyperglycaemia, indicating a potential adaptation of *β*-cell mass in response to insulin resistance [35]. Ectopic expression of mouse Pax4 in either human or rat islets as well as in the mouse insulinoma MIN6 cell line conferred protection against cytokine-mediated cell death and promoted islet cell proliferation [34, 39]. Interestingly, a diabetes-linked mutant variant R121W identified in the Japanese population was less efficient in protecting human islets against cytokines [34]. Supporting the role of Pax4 in survival/maintenance of *β*-cell mass, repression of Pax4 in the rat insulinoma INS-1E cell line and in hematologic cell lines that express high levels of the transcription factor provoked apoptosis [36, 40]. More recently, conditional overexpression of Pax4 in adult *β*-cells was shown to protect transgenic animals against STZ-induced hyperglycemia and isolated islets against cytokines, while animals expressing the mutant R121W variant were susceptible to developing hyperglycemia and *β*-cell death by both treatments. These antiapoptotic effects were shown to be mediated by increased expression of the antiapoptotic gene *bcl-2* and downregulation of the NF-*κ*B pathway. Consistent with a role for Pax4 in *β*-cell replication, the cell cycle-dependent kinase, cdk4 was increased in Pax4 overexpressing islets and promoted the proliferation of a Pdx1-positive subpopulation [41]. Together, these studies suggest that Pax4 functions as a survival and proliferation gene allowing mature islets to expand in response to physiological cues. 

An additional astonishing regenerative property of Pax4 that was recently uncovered is its capacity to reprogram *α*-cells to *β*-cells. Indeed, using an elegant Cre/LoxP approach in which the Cre recombinase was under the transcriptional control of the *glucagon* gene promoter, Collombat et al. showed that forced expression of Pax4 in developing *α*-cells induced a phenotypic switch towards *β*-cells. This was accompanied by an increase in islet size and in the quantity of insulin positive cells with a concomitant decrease in *α*-cell number. Remarkably, the conversion and thus decrease in the *α*-cell population resulted in neogenesis of new *α*-cells from duct-associated progenitor cells in adult animals. Nonetheless, these new replenished *α*-cells were continuously converted to *β*-cells due to Pax4 ectopic expression. Regeneration of the *β*-cell mass by aberrant expression of Pax4 in *α*-cell was able to transiently rescue hyperglycemia in young animals rendered diabetic by chemical treatment [42]. The mechanism by which Pax4 achieves reprogramming of *α*- to *β*-cells is thought to occur through the antagonistic effect of Pax4 on Arx, a key transcription factor involved in *α*-cell lineage commitment and subsequent mature function. It will be of great interest to determine whether the conditional and selective expression of Pax4 in mature *α*-cells using an inducible and reversible system will promote conversion to *β*-cells and whether repression of Pax4 will revert these cells back to *α*-cells. In this context, two independent studies have recently provided elegant evidence for the replenishment of the *β*-cell mass through *α*-cells conversion subsequent to severe pancreatic damage. Using an animal model in which the diphtheria toxin receptor was expressed specifically in *β*-cells, Thorel and colleagues obtained a near total *β*-cell ablation through administering of diphtheria toxin (DT) while maintaining all other endocrine cells intact [43]. This approach allowed the investigators to examine the contribution of remaining endocrine cell subtypes to putative *β*-cell regeneration. Using an inducible, tamoxifen-dependent Cre/Lox-based lineage tracing system, the authors concluded that following *β*-cell ablation, regeneration stemmed predominantly from a non-*β*-cell source. The irreversible labeling of *α*-cells showed that under approximately 99% *β*-cell loss, bihormonal cells expressing both insulin and glucagon were observed which ultimately generated single-hormone insulin positive cells. It is worth noting that the severity of the damage greatly influenced *α*-to-*β*-cell conversion, since this transdifferentiation was not observed under approximately 95% destruction. Why such reprogramming mechanisms are not observed by slightly lower amounts of *β*-cell loss remains to be understood. Consistent with these results, an independent study also claims to have observed pancreatic *β*-cell neogenesis by direct conversion of mature *α*-cells subsequent to pancreatic duct ligation (PDL) coupled to alloxan treatment. In this study, an astonishingly rapid *α*- to *β*-cell transdifferentiation was detected, resulting in the formation of new islets within 2 weeks. Nonetheless, treated mice never achieved normoglycemia. The authors proposed that the latter was most likely due to the profound and continuing inflammation and disruption of normal organ homeostasis that occurred following PDL/alloxan treatment [44]. An alternatively explanation which is not addressed could also be that the newly formed insulin-positive cells are nonfunctional *β*-cells. Although this study substantiates the work of Thorel and colleagues [43], no genetic lineage-tracing experiments were performed to clearly ascertain that *α*-cells were the predominant source of new *β*-cells. In this context, it is intriguing that although there was a greater rate of *β*-cell replication as compared to *α*-cell replication (6% versus 5% at 7 days after treatment), the authors excluded these residual *β*-cells as a potential source for regeneration in this particular injury model. Thus, whether a *bona fideα*- to *β*-cell reprogramming occurs in the PDL/alloxan model remains to be clearly established, and key factors driving this process have yet to be identified. In this context, determining whether Pax4 is implicated in the conversion of mature *α* to *β*-cells in these *in vivo* models of *β*-cell regeneration will be of great interest. Indeed, a recent study has challenged the role of Pax4 on the transdifferentiation of *α*- to *β*-cells, pointing to Men1/Menin as the main factor driving this process. In this case, it was found that specific ablation of Men1 in *α*-cells triggered reprogramming towards *β*-cells with the subsequent development of insulinoma [45]. Although Men1 could have a role in *α*-cell plasticity, it is currently difficult to discriminate whether this is a specific effect or only related to tumorigenesis. Independently, the identification of signaling molecules that trigger reprogramming of *α*-cells may offer new therapeutic tools for the treatment of DM.

Taken together, Pax4 certainly fulfills all requirements as a first-class target candidate for the development of innovative *β*-cell regenerative therapies for the treatment of DM. Indeed Pax4 (1) increases *β*-cell survival in response to metabolic stresses (2) stimulates proliferation, and (3) promotes conversion of *α*- to *β*-cells. Nonetheless, it is important to note that long-term expression of Pax4 may not be beneficial for *β*-cells as the cells revert to a progenitor phenotype thereby losing their capacity to secrete insulin in response to glucose [41]. Thus, identifying small molecules or factors that transiently activate Pax4 in either *α*- or *β*-cells will be instrumental for the development of novel antidiabetic treatment.

## 5. The (Orphan) Nuclear Receptor LRH-1

LRH-1 is a member of the NR5A2 subfamily of nuclear receptors which is predominantly expressed in the liver, [46], exocrine pancreas [47, 48], intestine [49], and ovaries [50]. In these tissues, LRH-1 regulates expression of genes involved in cholesterol and bile acid metabolism as well as steroidogenesis [51]. LRH-1 was also found to attenuate the acute phase response (APR) that is triggered in response to inflammatory cytokine signaling in the liver thereby preventing cell death [52]. Recently, human, mouse, and rat islets were shown to express LRH-1. More importantly, LRH-1 was found to partially convey the beneficial effects of estrogens on *β*-cell survival. Pharmacological studies using activators as well as inhibitors of estrogen receptors revealed that LRH-1 expression was predominantly induced through activation of Estrogen Receptor (ER)*α*. Repression of LRH-1 by RNA interference abrogated the protective effect conveyed by estrogen on islets against cytokines. Furthermore, previous studies have demonstrated that aberrant expression of LRH-1 is associated with the growth of intestinal tumors as well as with the proliferation of pancreatic and hepatic cell lines [49, 53]. However, the latter finding is in disagreement with adenoviral-mediated overexpression of LRH-1 in human islets, which did not increase *β*-cell proliferation [54]. LRH-1 appears to promote cell replication by stimulating expression of cyclinD1 and E1, a process that involves a crosstalk with the *β*-catenin/Tcf4 signaling pathway [49]. Low levels of *β*-catenin in mature islet may provide an explanation for the lack of LRH-1 effect on cell proliferation [54]. In contrast, LRH-1 was found to confer protection against cytokine- and STZ-induced apoptosis. The steroidogenic enzymes CYP11A1 and CYP11B1 that are involved in glucocorticoid biosynthesis were increased in transduced islets. As these steroids are potent immunosuppressors, glucocorticoids in a paracrine manner may mediate the protective effect of LRH-1 [54]. Of note, glucocorticoids were shown to be diabetogenic and to hamper islet function *in vivo* [55, 56]. These findings led to their exclusion from immunosuppressive regimens given to patients subsequent to islet transplantation [7]. However, a recent study demonstrated that exogenous glucocorticoids have potent anti-inflammatory properties on human islets. Furthermore, although glucocorticoid-treated human islets exhibited a rapid reduction in glucose-induced insulin secretion observed within 24 hours, these islets performed considerably better than control islets in long-term culture [57]. Consistent with the beneficial impact of glucocorticoids on islet integrity, a recent study demonstrated that optimal elevation of glucocorticoids in *β*-cells was a compensatory mechanism that prevented high-fat diet-induced *β*-cell failure [58]. Thus, in contrast to previous beliefs, low levels of glucocorticoids generated endogenously by factors such as LRH-1 may be advantageous to preserve *β*-cells against metabolic aggressions.

Given the importance of LRH-1 in regulating several key metabolic pathways including lipid metabolism as well as in modulating cellular homeostasis (cell proliferation and survival), attempts to regulate its activity could be of therapeutic value for the treatment of DM. In this context, although LRH-1 is considered an orphan receptor that possesses constitutive activity, small endogenous phospholipid were shown to bind the ligand pocket domain of the protein and to increase its activity [59]. Taking advantage of this knowledge, a recent study demonstrated that administration of the phospholipids dilauroyl phosphatidylcholine (DLPC) decreased hepatic steatosis and improved glucose homeostasis in two mouse models of insulin resistance. These beneficial effects were predominantly mediated through increased hepatic LRH-1 activity. These results have led to a pilot human clinical study in USA to explore the effects of DLPC in pre-T2DM patients [60]. In parallel, it will be of interest to determine whether small molecule agonists of LRH-1 recently developed by Whitby and colleagues [61] can protect human islets against stressed-induced cell death and prevent onset of hyperglycemia in animal models of experimental autoimmune diabetes. The anti-inflammatory properties combined with the prosurvival effects of LRH-1 highlight the immense potential of this newcomer and rising star target for the treatment of both T1DM and T2DM.

## 6. Perspectives

**Figure 1:**
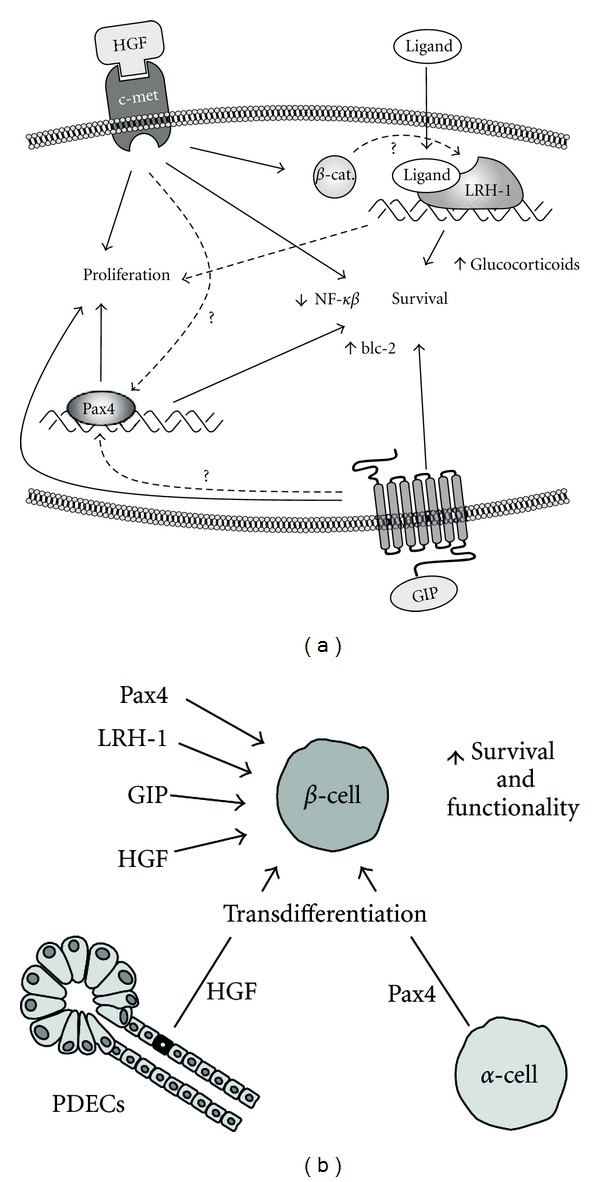
HGF, Pax4, GIP, and LRH-1 increase *β*-cell regeneration and preservation. Schematic representation of the putative interactions among HGF, GIP, Pax4, and LRH-1 that impact *β*-cell survival and expansion. (a) These factors regulate common pathways and targets such as NF-*κ*B and bcl-2 that exert beneficial effects on *β*-cells. (b) HGF and Pax4 promote transdifferentiation from other pancreatic cells to *β*-cells in addition to enhancing *β*-cell survival and function.

DM is now considered one of the most common noncommunicable diseases in the world causing 5% of all deaths per year. Regardless of its aetiology, the end point of DM is *β*-cell death. Therefore, new therapies should aim at restraining *β*-cell death and promote *β*-cell performance in patients in order to improve blood glucose without treatment-derived side effects. Currently, a potpourri of growth factors and transcription factors have been highlighted for their ability to preserve and/or increase *β* cell mass. The hierarchy or specific networks connecting them together and value in treating diabetic patients are yet to be established. Most likely these factors will have to be used in combination to optimize viability of *β*-cells as well as to increase the replication capacity. Nonetheless, as current published data point to a declining capacity of *β*-cells to replicate with age [62, 63], efforts should focus on characterizing factors capable of preserving and protecting the *β*-cell cell mass under pathophysiological situations. In this context, HGF, GIP, Pax4, and LRH-1 in various permutations may hold the key to a successful regenerative therapy applied to DM. Indeed, these factors appear to either complement or share common signaling pathways and downstream targets resulting in increase islet viability and performance (Figure 1). For instance, HGF and Pax4 both inhibit the NF-*κ*B pathway leading to increased *β*-cell survival. Similarly, GIP and Pax4 enhance expression of the antiapoptotic gene bcl-2. As we have previously demonstrated that GLP-1 stimulates Pax4 expression in human islets [35], it will be of interest to determine whether GIP also increases Pax4 expression resulting in the downstream activation of the *bcl-2* gene. Consistent with this possibility, both GIP-mediated *β*-cell protection and GLP-1-conveyed increases in Pax4 are relayed by the PI3K signaling pathway [27, 35]. HGF combined with small LRH-1 agonists may be sufficient to stimulate human islet *β*-cell expansion. Indeed, HGF was shown to provoke the release of *β*-catenin from c-met with its subsequent translocation to the nuclei [14]. Accumulation of nuclear *β*-catenin may reach sufficiently high levels to interact with LRH-1 and activate downstream target gene such as *cycd1* [49]. In addition, both LRH-1 and HGF have been shown to decrease inflammation, an important mediator of cell death in DM [18, 54]. Finally, HGF and Pax4 have the additional property to promote transdifferentiation of PDECs and *α*-cells into *β*-cells. This could be extremely beneficial as an alternative method to replenish a functional *β*-cell mass due to the limited replication capacity of *β*-cells. It is also interesting to note that *α*-cells appear less susceptible to autoimmune attack with an apparent increase in the number of *α*-cells in Type 2 diabetic subjects. In this context, a combined HGF/c-met, Pax4, and GIP therapy could be optimal: *β*-cell protection with increased proliferation as well as the generation of new *β*-cells from *α*-cells. Nonetheless one important consideration to take into account is that regeneration and tissue responses are very different under various contexts, such as the severity of the injury or the age of disease onset. Furthermore, vigilance is of essence also to restrain the potential deregulated cellular growth when using such factors. Thus, to be successful, this type of regenerative therapy requires intervention at a threshold point at which *β*-cells are still present and that the pancreas retains some regenerative plasticity. Alternatively, these factors could also be useful for optimizing islet transplantation. Indeed, increased islet isolation yields and posttransplantation islet performance and survival could be feasible using a combination of HGF, GIP, Pax4, and LRH-1. The latter approach would reduce the number of islets required for transplantation and improve long-term islet function. 

Islet *β*-cell regeneration is a fast moving field in which great advances can be achieved in the next few years with promising potential for the treatment of DM. The next step is to elucidate the molecular mechanisms that intertwine HGF, GIP, Pax4, and LRH-1 together in promoting survival and rejuvenation of islet cells. These studies will most likely highlight additional factors that may become novel targets for regenerative therapies.
