# Corrigendum to “Islet *β*-Cell Mass Preservation and Regeneration in Diabetes Mellitus: Four Factors with Potential Therapeutic Interest”

**DOI:** 10.1155/2019/3281921

**Published:** 2019-06-02

**Authors:** Jose Manuel Mellado-Gil, Nadia Cobo-Vuilleumier, Benoit R. Gauthier

**Affiliations:** Pancreatic Islet Development and Regeneration Unit, Department of Stem Cells, CABIMER-Andalusian Center for Molecular Biology and Regenerative Medicine, Avenida Américo Vespucio, Parque Científico y Tecnológico Cartuja 93, 41092 Sevilla, Spain

In the article titled “Islet *β*-Cell Mass Preservation and Regeneration in Diabetes Mellitus: Four Factors with Potential Therapeutic Interest” [1], a grant number was missing. The Acknowledgments section should be updated as follows:

The authors acknowledge the financial support of the Consejeria de Salud, Junta de Andalucía (PI-0727/2010 to B. Gauthier); the Spanish Ministry of Science and Innovation, Instituto de Salud Carlos III cofinanced by European Funds for Regional Development (FEDER) (PI10/00871 to B. Gauthier); the Fundacion Publica Andaluza Progreso y Salud (to B. Gauthier and J. Mellado-Gil); and the Consejería de Innovación, Ciencia y Empresa, Junta de Andalucía (CTS-6359 to B. Gauthier).
